# A differentiable variational model for structural self-contact and fracture

**DOI:** 10.1007/s00366-026-02285-6

**Published:** 2026-02-19

**Authors:** Mirko Ciceri, Charlie Aveline, Dilaksan Thillaithevan, Robert Hewson, Matthew Santer

**Affiliations:** Department of Aeronautics, Imperial College London, Exhibition Rd, South Kensington, London, SW7 2AZ UK

**Keywords:** Phase field, Fracture, Third medium contact

## Abstract

Numerical modelling of structural self-contact and crack propagation presents significant challenges due to the inherently discontinuous and non-differentiable nature of the underlying physical phenomena. Traditional contact models demand explicit definition and tracking of contact points, while fracture models often rely on predefined crack initiation sites, sharp interfaces, and re-meshing. This study introduces a novel framework that overcomes these limitations within a unified and numerically stable variational formulation. The contact phenomenon is described through the hyperelastic third medium contact model and fracture is represented by a phase field. Structures are embedded in a third medium that stiffens under compression, enabling the transfer of forces between structural members. Crack propagation occurs in regions in which it is energetically favourable for the system to evolve toward a fully damaged state, specifically where the critical energy release rate is exceeded. Careful treatment is required when coupling the two phenomena, particularly concerning the void material behaviour. This work presents an efficient and differentiable numerical model that captures both nonlinear phenomena within a unified framework. This framework will allow designers and engineers to efficiently analyse complex nonlinear structural behaviours, previously requiring separate models that involved pre-defined crack initiation sites and contact points. Lastly, the differentiable nature of the model facilitates straightforward future integration into topology optimisation pipelines, providing designers the ability to intentionally design for and leverage self-contact interactions and material failure as functional, performance-enhancing features.

## Introduction

The consideration of fracture and contact phenomena are crucial when assessing structural integrity, material failure and the interaction of deformable bodies in engineering applications. The discrete and discontinuous nature of these events demands explicit simulation methods, which often require prior selection of failure zones and the prediction of contact pairs. This process is not straightforward for complex structures and severely hinders systematic and automated simulation. Independent variational models of fracture and internal self-contact have been proposed, but have not been considered in a unified computational framework. Variational formulations avoid the need for pre-defined contact pairs, and crack nucleation sites. This is particularly useful when pre- or post-failure structural boundaries or contact points, are not known a priori. In this work we present a unified formulation for self-contact and material failure. Fracture is tracked using a phase field (PF) model, which is outlined in Sect. 1.1, and self-contact is introduced via the third medium contact (TMC) model, as discussed in Sect. 1.2.

### Fracture

Due the inherently nonlinear and discontinuous nature of fractures, few analytical solutions for crack problems exist in literature. Those based on linear elastic fracture mechanics (LEFM) are limited to simple geometries and loading conditions [1]. Numerical modelling through finite elements provides a viable approach to studying crack propagation. Several computational methods have been proposed, including the discrete cracking method, the extended finite element method (XFEM), phase field, peridynamics (PD), particle finite element method (PFEM), each with distinct strengths and limitations. A review on the main numerical methods for fracture can be found in [2]. The phase field method has been adopted for failure modelling due to its versatility and its variational nature. It has gained popularity across a wide range of scientific disciplines such as material science, chemistry, biophysics and mechanical/civil engineering. This method enables a continuous description of physical discontinuities, eliminating the need for explicit interface tracking and handling of singularities.

In the PF model for fracture, cracks are represented by a diffuse scalar field, $\alpha \in [0,1]: \Omega \rightarrow \mathbb {R}$, where 0 and 1 represent intact and fully damaged material, respectively, and $\Omega$ is the modelling domain. Crack nucleation and propagation results from energetic considerations within the fixed mesh representation. Although rooted in Griffith’s energetic formulation [3], PF models go beyond it by naturally handling crack nucleation, branching, and merging, as well as predicting complex crack trajectories. The phase field method for fracture requires the simultaneous solution of the displacement field $\boldsymbol{u}$ and the phase field variable $\alpha$. The associated total energy functional is typically non-convex due to the nonlinear dependence of material stiffness on the phase field. Therefore, the solution of this coupled, highly nonlinear system requires careful numerical strategies, often involving incremental loading, regularization, and robust solvers.

The pioneering work on the development of a variational approach to fracture can be attributed to Francfort and Marigo in [4] and Burdin *et al. * in [5], who provided rigorous mathematical interpretations of the regularised crack propagation problem. Their reformulation avoids the need for explicit line integral definitions of cracks, whose lengths are not known in advance, through a global energy minimisation. Amor *et al. * in [6] proposed an anisotropic version of the model with the introduction of an additive strain energy decomposition. This is necessary for distinguishing between the energy related to tension, responsible for crack opening, and compression, which does not contribute to crack propagation. Miehe *et al. * in [7] proposed a thermodynamically consistent formulation for decoupling the coupled nonlinear and non-convex problem into two independent linear and convex problems. This split allowed the adoption of a staggered solution strategy, which improves solver stability at the cost of added computational expense. Furthermore, their work introduces a history parameter which accounts for the irreversible nature of material failure. Ambati *et al. * proposed a further simplification that considers the energy split only in the phase field equation [8]. This simplifies the displacement equation and renders the model more computationally efficient without a loss in accuracy. Recent research highlights the length scale independency of the model, where the fracture process zone and structural response become independent of the phase field regularization length, drawing analogies to cohesive zone models as shown in [9], with the goal of providing an accurate description of quasi-brittle fracture. Further advancements were made by the same authors with the introduction of the generalized phase field cohesive zone model ($\mu$PF-CZM) [10]. Kumar *et al. * [11] revisited phase field formulations by incorporating an explicit nucleation stress criterion within the variational framework, improving physical consistency independently of regularization effects. A critical assessments by Lopez–Pamies *et al. * [12] highlight limitations of classical variational phase field models, particularly their inability to predict fracture nucleation in brittle elastic materials due to the absence of explicit material strength parameters. PF models have been extended to a wide range of applications including dynamic fracture [13, 14], plasticity [14, 15], fatigue [16], hyperelastic materials [17], corrosion [18, 19], hydrogen embrittlement [20], composites [21], topology optimisation [22] and many others. Machine learning has also been integrated with phase field fracture models. Manav *et al. * [23] developed a physics-informed neural network approach using the deep Ritz method to enhance predictions of crack nucleation, propagation and kinking. For a comprehensive review of phase field models for fracture, covering both theoretical foundations and computational implementations, the reader is referred to [24].

The primary limitations of PF models are their computational expense and stability issues. Extremely fine mesh resolution is needed along the crack propagation path to recover the sharp crack geometries. If the crack nucleation locations and crack path are not known in advance, the use of uniform fine mesh resolutions over the entire domain severely increases the computational expense. The instantaneous and uncontrolled nature of crack propagation makes the numerical solution unstable, requiring problem-specific, manual fine-tuning of solution strategies. Several approaches have been proposed to address these challenges, such as adaptive time stepping and mesh refinement [25–27]. While these methods help reduce computational cost and improve robustness, they often introduce additional complexity. Similarly, staggered and quasi-Newton solvers [28–30], while enhancing convergence, do not resolve the inherent instabilities in the highly nonlinear, non-convex energy landscape. Thus, despite progress, efficient, stable, and fully automated simulations of complex fracture phenomena remain an ongoing challenge.

### Contact

Contact mechanics has been extensively studied in the context of the finite element method, as highlighted in [31]. Modelling contact is essential, especially when dealing with structures undergoing large deformations to prevent non-physical self-penetration. Various approaches for modelling contact exist, including node-to-node, node-to-segment, and mortar methods [32]. These methods all require the introduction of inequality constraints within the global energy minimisation, which are typically introduced via penalty methods, Lagrange multipliers and barrier functions, which add further numerical complications.

A variational alternative to these traditional methods is the frictionless third medium contact approach, originally introduced by Wriggers *et al. * in [33]. This approach circumvents the need for inequality constraints by exploiting anisotropic modelling, inherent in most hyperelastic formulations. Contacting bodies are embedded in a highly compliant medium (the third medium), which becomes increasingly stiff under large compressions and allows stresses to be transferred between structural members. Unlike classical methods, this model cannot describe net transitions from zero to finite contact stress. The main challenge of this model lies in defining a numerically stable constitutive law for the contact medium undergoing large deformations without impacting the behaviour of the solid material. Although initially overlooked, as reported in [34], the third medium approach has recently gained renewed interest for its potential in topology optimisation problems, where contact boundaries are not predefined [35–37]. In such scenarios, modelling voids is inherent in the procedure and smoothness of contact allows for the computation of gradients. The TMC method is employed in this work due to its variational nature and differentiable description.

The combined treatment of contact and fracture phenomena can be achieved using conventional methods however, this generally comes at the expense of differentiability. In [38], a subroutine implemented in the commercial software Abaqus in conjunction with a hard contact surface-to-surface interaction, is used to model the three-dimensional fracture of gears under contact. Although this work demonstrates the feasibility of extending conventional algorithms to 3D and incorporating contact effects, the reliance on hard contact and the need of defining contact pairs inevitably hinders differentiability. Other significant efforts to address contact within phase-field fracture models are presented by Fei et al. [39] and Wheeler et al. [40]. These approaches are restricted at the self-contact representation along crack surfaces, and include frictional stick–slip behaviour within the diffuse crack region. Typically, they rely on augmented Lagrangian or penalty-based formulations to prevent interpenetration of crack faces and to enforce irreversibility constraints on crack evolution. More recently, peridynamics has emerged as a viable tool for simulating fracture under dynamic and complex conditions. Unlike phase field models, peridynamics represents interactions via pairwise forces between material points, inherently accommodating discontinuities and crack propagation without additional criteria [41]. Recent studies have further enhanced peridynamic models by incorporating contact detection algorithms. Advanced node to node strategies enable the identification of potential contact pairs and the computation of contact forces through penalty-based formulations, achieving results consistent with classical contact mechanics [42–44]. Although advances in contact detection algorithms improve the identification of interacting points, the use of penalty-based formulations introduces non-smooth forces, making the model difficult to differentiate.

The present work proposes a unified variational and differentiable computational framework that integrates fracture and self-contact, two phenomena traditionally treated independently. The coupling of phase field and third medium contact introduces complex interactions, particularly in regions transitioning from solid to void. To address this, suitable interpolation functions are designed to ensure smooth and physically consistent behaviour across evolving material domain. A central challenge lies in ensuring numerical stability, which is addressed through the introduction of a solution strategy tailored to the coupled problem. The remainder of this paper is structured as follows: Sect. 2 outlines the numerical modelling framework for coupling hyperelastic TMC and phase field models. The numerical implementation within a FEM environment, along with the solution technique employed is provided in Sect. 3. Section 4 presents the numerical validation of the model against two classical fracture mechanics numerical results, with the conclusions presented in Sect. 5.

## Modelling approach

### Constitutive law

**Fig. 1:**
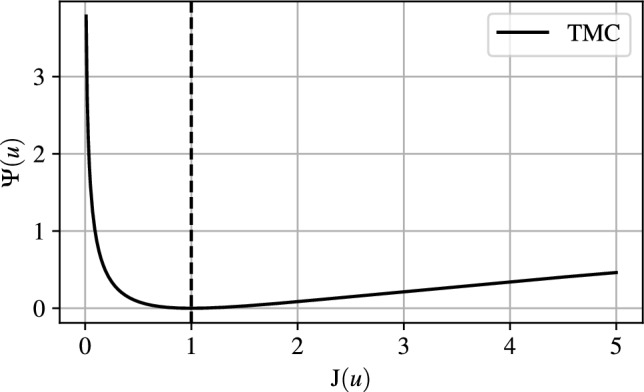
Hyperelastic strain energy density as a function of the determinant of the deformation gradient

The TMC model [33] is utilised in this work to capture internal self-contact. The material strain energy density ${\Psi }(\boldsymbol{u})$ is expressed by a hyperelastic Neo-Hookean model with the addition of a void regularisation term

1 $$\begin{aligned} {\Psi }(\boldsymbol{u}) = \underbrace{\bar{\Psi }(\boldsymbol{u})}_{\text {Hyperelastic Neo-Hookean Term}} + \underbrace{\bar{\Phi }(\boldsymbol{u})}_{\text {Void Regularisation Term}} \end{aligned}$$

where $\boldsymbol{u}$ is the displacement vector. The strain energy density function of the solid material is defined as

2 $$\begin{aligned} \bar{\Psi }(\boldsymbol{u}) = \underbrace{\frac{K}{2} (\ln (\det (\textbf{F}))^2}_{{\Psi }^{Vol}} + \underbrace{\frac{G}{2} \left( \det (\textbf{F})^{-2/3} ||\textbf{F}||^2 - 3 \right) }_{{\Psi }^{Dev}} \end{aligned}$$

in which *K* and *G* represent the bulk and shear modulus respectively, and $\textbf{F} = \textbf{I} +\nabla \boldsymbol{u}$ is the deformation gradient tensor. The term $J = \det (\textbf{F})$ represents the local volume change during deformation and is a key component of the TMC model. In particular the transfer of forces between structural members is enabled since $\Psi \rightarrow \infty$ as $J \rightarrow 0$, as shown in Fig. 1 in a 1D representation.

In traditional hyperelastic modelling the limit $J \rightarrow 0$ represents a limitation as it introduces numerical instabilities. In TMC this condition is exploited and it is integral part of the solution. Therefore, when severely deformed, void elements become stiffer than the solid phase and enable the transfer of forces between solid members. While the severe deformation of void elements is necessary for modelling contact, it results in the presence of highly distorted elements. To prevent numerical instabilities, different regularisation terms have been proposed. These terms often consider the energy associated with higher order deformations. A detailed formulation of the void regularisation term was presented in [45] and can be defined as

3 $$\begin{aligned} \bar{\Phi }(\boldsymbol{u}) = k_r \frac{1}{2}\left( \mathbb {H}_{\boldsymbol{u}} \vdots _{} \mathbb {H}_{\boldsymbol{u}} - \frac{1}{Tr (I)} \mathbb {L}_{\boldsymbol{u}} \cdot \mathbb {L}_{\boldsymbol{u}} \right) \end{aligned}$$

where $k_r = \bar{k_r} L^2 K$ is a dimensional scaling parameter, L is a characteristic structural dimension and $\bar{k_r}$ is an adequately small regularisation value. Lower $\bar{k_r}$ values reduce the stiffening of the void region, whereas higher values stabilise the problem. The constant $\bar{k_r}$ must be chosen small enough to regularise the problem in the void domain without interfering with the behaviour of the solid structure. This scaling allows working with a dimensionless quantity $\bar{k_r}$ with more general validity as shown in [46]. The term $\mathbb {H}_{\boldsymbol{u}} = \partial ^2 u / \partial x_i \partial x_j$ represents the Hessian of the displacement field. This term introduces strain energy where the mesh is highly deformed but it is overly conservative for specific higher order deformation modes including bending and quadratic compression. The subtraction of the term $\mathbb {L}_{\boldsymbol{u}} = \partial ^2 u / \partial x_i^2$ that represents the Laplacian operator, serves as a regularisation reduction for the specific over-regularised modes. This regularisation method has been shown to be particularly effective to handle 3D problems with coarse meshes [45]. Other terms can be selectively multiplied by $\bar{\Phi }$ in Eq. (3) with the aim of further reducing the influence of the regularisation term on the solid structural behaviour. A void indicator function $\mathcal {I} \in [0,1]: \Omega \rightarrow \mathbb {R}$, where 0 and 1 represent solid and void material, respectively enables the regularisation of void elements only. A term $\exp {(-5 \det (\textbf{F}))}$, that results from numerical experiments, can be included as well and it leads to decaying regularisation for elements that are not severely compressed or in tension [46].

The main limitation of this class of regularisation is the need for higher order elements for $\boldsymbol{u}$, as $\bar{\Phi }$ requires the computation of $\mathbb {H}_{\boldsymbol{u}}$. Recently Wriggers proposed an alternative regularisation scheme that does not employ higher order elements, but instead requires the solution of additional variables [34] and exploring its implementation will be subject of future work.

### Fracture modelling

**Fig. 2:**
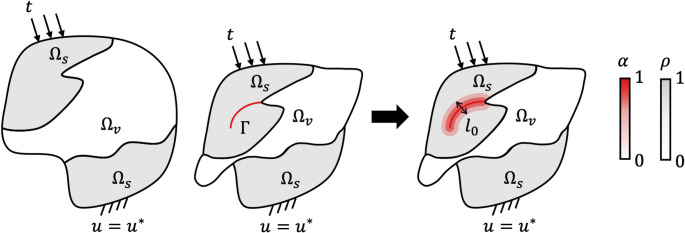
Phase field regularisation in the context of TMC, with $\Omega _s$ solid region, $\Omega _v$ void region and *L* example of a characteristic structural dimension

A rate-independent AT-2 phase field model is adopted to describe brittle fracture. The problem is formulated as a global energy minimisation where crack propagation results from the energy balance between stored and surface energy terms

4 $$\begin{aligned} \Pi (\boldsymbol{u},\Gamma ) = \underbrace{\int _\Omega \Psi (\boldsymbol{u}) d\Omega }_{\text {Stored energy}} + \underbrace{G_c \int _{\Gamma } d\Gamma }_{\text {Surface energy}} \end{aligned}$$

and where $\Pi$ represents the total potential energy, given by the sum of the stored energy and surface energy related to the formation of cracks. In this work the stored energy term considers the hyperelastic TMC formulation presented in Eq. (1). The surface energy is dependent on $G_c$, the critical energy release rate, and the integration over a discontinuous and unknown crack domain $\Gamma$, as depicted in Fig. 2. For numerical stability, the problem is regularised, with cracks represented by diffused bands of finite thickness $l_0$.

The PF variable is approximated by an exponential profile, $\alpha (\textbf{x}) = \exp {\left( -\frac{|\textbf{x}|}{l_0}\right) }$, where $\textbf{x}$ represents a spatial coordinate, which localizes around the crack. As $l_0 \rightarrow 0$, this profile approaches a sharp discontinuity, corresponding to a discontinuous crack topology. Under this approximation, the total energy functional $\Pi$ can then be reformulated as

5 $$\begin{aligned} \Pi (\boldsymbol{u},\alpha ) = \underbrace{\int _\Omega g(\alpha ) {\bar{\Psi }}(\boldsymbol{u}) d\Omega }_{\Pi _{\text {{stored}}(\boldsymbol{u},\alpha )}} + \underbrace{\int _\Omega \bar{\Phi }(\boldsymbol{u})d\Omega }_{\Pi _{\text {reg}} (\boldsymbol{\text {u}})} + \underbrace{ \frac{G_c}{c_w} \int _{\Omega } \left[ \frac{w(\alpha )}{l_0} + l_0 |\nabla \alpha |^2 \right] d\Omega }_{\Pi _{\text {surface}}(\alpha )} \end{aligned}$$

where $\alpha$ is the order parameter or crack phase-field and the limits $\alpha = 1$ and $\alpha = 0$ represent the fully degraded and the fully intact material phases, respectively. The function $g(\alpha )$ is the degradation function, responsible for the energy reduction while $w(\alpha )$ is the crack geometric function and $c_w:= 4 \int _0^1 \sqrt{w(\beta )} \ d\beta$ is a regularization constant ensuring $\Gamma$-convergence to the sharp-interface fracture energy, where $\beta \in [0,1]$ parametrises the damage transition.

The evolution of $\alpha$ is driven by the minimisation problem in Eq. (5). Cracks result from the balance between stored energy and the energy required to create new fracture surfaces, leading to the gradual localised energy reduction and consequently to the propagation of diffused cracks in the material.

#### Energetic decomposition

The formulation in Eq. (5) is symmetric with respect to $\bar{\Psi } (\boldsymbol{u})$ and would lead to crack propagation in tension and compression. To circumvent this deficiency of the model, an energetic split is employed. Different splits have been proposed in the literature with respect to the constitutive material formulation used and the desired failure modes. Considering the hyperelastic, TMC material definition a volumetric-deviatoric split

6 $$\begin{aligned} \Psi ^+(\boldsymbol{u}) = {\left\{ \begin{array}{ll} {\Psi }^{Vol} + {\Psi }^{Dev} & \text {if } J\ge 1 \\ {\Psi }^{Dev} & \text {if } J<1 \end{array}\right. } \end{aligned}$$

is proposed in which the tensile energy contribution is determined by the determinant of the deformation gradient, *J*. Values below unity, indicate the structure is experiencing local compression and only the deviatoric energy component, ${\Psi }^{Dev}$, is included. Where the material experiences tension both the volumetric (${\Psi }^{Vol}$) and deviatoric components of energy are included.

Alternative energetic decompositions [47] could also be employed without altering the problem structure. Since the focus of this manuscript is on the coupling of phase field with TMC rather than on the specific fracture energy decomposition the Vol-Dev split has been proposed for convenience and clarity.

#### Crack irreversibility

In the proposed configuration $\alpha$ is only related to the energy levels in the domain and therefore if the load is removed, $\alpha$ would decrease leading to fracture recovery. Many techniques exist to respect the irreversibility constraint $\dot{\alpha }\ge 0$ and consequently prevent damage recovery. For a review of these methods the reader is referred to [48]. A widely adopted technique to avoid the introduction of an inequality constraint is the use of a history parameter. It is used to discretise the irreversibility condition such as $\alpha _n \ge \alpha _{n-1}$ where the subscript stands for the *n*-th load discretisation step. This parameter can be defined as

7 $$\begin{aligned} \mathcal {H}_n := \max \left( \mathcal {H}_{n-1}, \Psi ^+_n(\boldsymbol{u})\right) \end{aligned}$$

where $\mathcal {H}_{n-1}$ is the largest strain energy experienced up to load step *n* and $\Psi ^+_n(u)$ is the current portion of strain energy that contributes to damage.

Following the hybrid formulation proposed by Ambati in [8], the global strain energy $\bar{\Psi }(u)$ is considered in the stored energy, while only the component contributing to damage $\Psi ^+(\boldsymbol{u})$ is used in $\mathcal {H}_n$.

### Thermodynamic fluxes and weak formulation

The mechanical equilibrium of the system in its weak form is obtained by enforcing

8 $$\begin{aligned} \mathcal {R}(\boldsymbol{u},\alpha ,\delta \boldsymbol{u},\delta \alpha )=\delta \Pi (\boldsymbol{u},\alpha ) = 0 \quad \forall \delta \boldsymbol{u},\delta \alpha \end{aligned}$$

where $\delta \boldsymbol{u}$ and $\delta \alpha$ represent the variations of the displacement and the phase field.

The total energy functional comprises the hyperelastic stored energy, a second-order gradient regularisation term and the surface energy contribution associated with fracture, therefore its variation can be written as

9 $$\begin{aligned} \delta \Pi (\boldsymbol{u},\alpha )= \delta \Pi _{stored}(\boldsymbol{u},\alpha ) + \delta \Pi _{{reg}}(\boldsymbol{u}) + \delta \Pi _{surface}(\alpha ,\nabla \alpha ). \end{aligned}$$

By expressing the variation of the deformation gradient as $\delta \textbf{F}=\nabla \delta \boldsymbol{u}$, the corresponding contribution of the hyperelastic stored energy to the weak form is

10 $$\begin{aligned} \delta \Pi _{stored} = \int _\Omega \left[ g(\alpha )\,\frac{\partial \bar{\Psi }}{\partial \textbf{F}} : \nabla \delta \textbf{u} + g'(\alpha )\,\bar{\Psi }\,\delta \alpha \right] \, d\Omega \end{aligned}$$

and from this expression, the mechanical flux associated with the stored energy is identified as

11 $$\begin{aligned} & \textbf{P} = g(\alpha )\,\frac{\partial \bar{\Psi }}{\partial \textbf{F}} = g(\alpha )\left( K \,\ln (\det (\textbf{F}))\textbf{F}^{-T} \right. \nonumber \\ & \quad \left. + G \det (\textbf{F})^{-2/3}\textrm{dev}(\textbf{F} \textbf{F}^T)\textbf{F}^{-T}\right) \end{aligned}$$

which corresponds to the standard first Piola–Kirchhoff stress of the hyperelastic material [35] modulated by the degradation function $g(\alpha )$. The thermodynamic force associated with the phase field contribution of the stored energy is

12 $$\begin{aligned} Y_{stored} = g'(\alpha )\,\bar{\Psi } \end{aligned}$$

in which, following the irreversibility condition in Eq. (7), $\bar{\Psi }$ is replaced by the history variable $\mathcal {H}_n$. Similarly, the regularisation term contributes to the weak form through its first variation with respect to the displacement field. Following [45], this variation is given by

13 $$\begin{aligned} \delta \Pi _{\textrm{reg}} = \int _\Omega k_r \left( \mathbb {H}_{\textbf{u}} \vdots \mathbb {H}_{\delta \textbf{u}}- \frac{1}{\operatorname {Tr}(\textbf{I})} \mathbb {L}_{\textbf{u}} \cdot \mathbb {L}_{\delta \textbf{u}} \right) d\Omega . \end{aligned}$$

Its role is purely numerical and, if scaled correctly, its energetic contribution remains several orders of magnitude smaller than the stored hyperelastic energy, in line with the observations reported in [36].

The first variation of the energy required for the generation of new crack surfaces can be defined

14 $$\begin{aligned} \delta \Pi _{\textrm{surface}} = \frac{G_c}{c_w} \int _{\Omega } \left[ \frac{w'(\alpha )}{l_0}\,\delta \alpha + 2 l_0\, \nabla \alpha \cdot \nabla \delta \alpha \right] \, d\Omega , \end{aligned}$$

from which the thermodynamic forces conjugate to the local damage variable $\alpha$ and its gradient $\nabla \alpha$ can be obtained as

15 $$\begin{aligned} Y_\alpha = \frac{G_c}{c_w}\frac{w'(\alpha )}{l_0}, \qquad \boldsymbol{\xi } = \frac{2 G_c}{c_w} l_0 \nabla \alpha \end{aligned}$$

where $\boldsymbol{\xi }$ governs the diffusive regularisation of damage and combining the energetic contributions associated with $\delta \alpha$ we obtain the total driving force $Y = Y_{stored} + Y_{\alpha }$. Collecting all terms, the weak form reads

16 $$\begin{aligned} \begin{aligned}\mathcal {R}(\boldsymbol{u},\alpha ,\delta \boldsymbol{u},\delta \alpha )= \int _\Omega \left[ \left( \boldsymbol{P} : \nabla \delta \boldsymbol{u}\right) + k_r \left( \mathbb {H}_{\boldsymbol{u}} \vdots _{} \mathbb {H}_{\delta \boldsymbol{u}} - \frac{1}{Tr (I)} \mathbb {L}_{\boldsymbol{u}} \cdot \mathbb {L}_{\delta \boldsymbol{u}} \right) \right] d\Omega \, \\+ \int _{\Omega } \left[ \left( g'(\alpha ) \mathcal {H}_n + \frac{G_c}{c_w} \frac{w'(\alpha )}{l_0} \right) \delta \alpha + \frac{2G_c}{c_w} l_0 \nabla \alpha \cdot \nabla \delta \alpha \right] d\Omega = 0 \end{aligned} \end{aligned}$$

in which $g(\alpha )$, $w(\alpha )$ and consequently $c_w$ define the coupling between displacement and phase field equation. In this work a classical AT-2 regularisation has been adopted in which $g(\alpha ) = (1-\alpha )^2$, $w(\alpha ) = \alpha ^2$ and $c_w = 2$. Other choices of degradation and dissipation functions exist in the literature. These are not shown in this paper since comparisons of these functions are outside the scope of this work. Nevertheless different formulations such as AT-1, fourth order formulations and cohesive zone models would require proper selection of phase field constitutive functions.

### Material interpolation

**Fig. 3:**
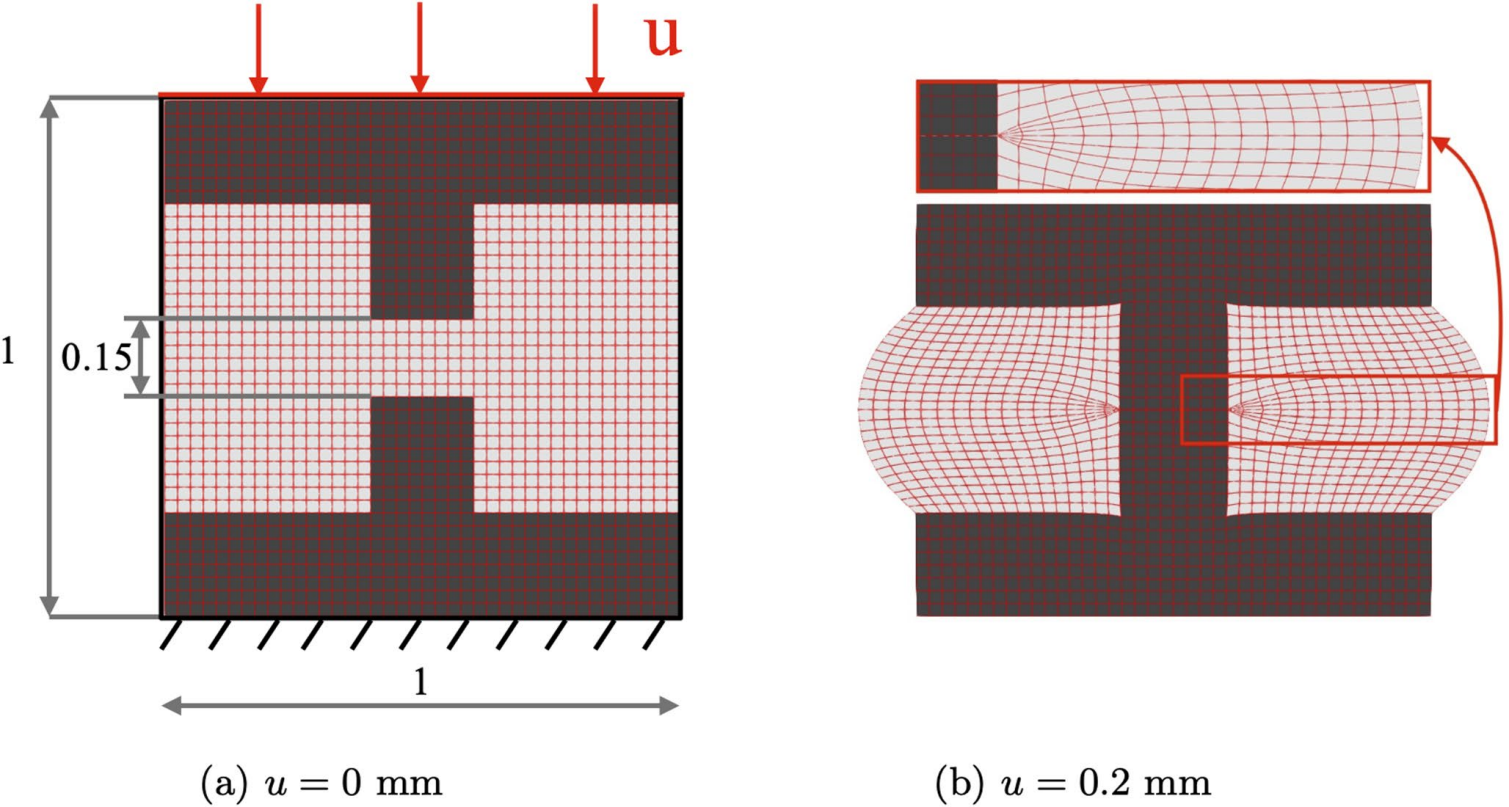
Deformation, loading conditions and geometrical dimensions of benchmark structure at discrete imposed displacement for $\gamma _0 = 1 \times 10^{-5}$

In this model internal structural boundaries are defined by a pseudo density function $\rho \in [0,1]: \Omega \rightarrow \mathbb {R}$. To each element in the domain a pseudo-density scalar has been assigned. Pseudo-density is a continuous function defining the transitions from void to solid and can be described by the material interpolation function

17 $$\begin{aligned} \gamma (\rho ) = \gamma _0 + (1-\gamma _0) \rho \end{aligned}$$

in which $\gamma _0$ represents the minimum value of $\gamma$, corresponding to the void properties. Each material property is obtained through the multiplication between $\gamma$ and the solid material property defined by the subscript 0 i.e. $K(\rho ) = K_0 \cdot \gamma (\rho ), G(\rho ) = {G}_0 \cdot \gamma (\rho )$. The value of $\gamma _0$ is critical, as it controls the void material stiffness. The void stiffness, $\gamma _0$, should be chosen to be as small as possible to prevent the introduction of parasitic forces yet big enough to prevent numerical instabilities. A benchmark structure, shown in Fig. 3, has been designed to test the influence of the parameter $\gamma _0$ [49].

**Fig. 4:**
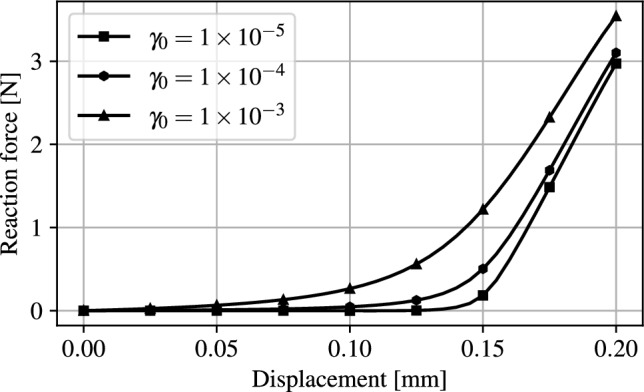
Force-displacement curve as a function of $\gamma _0$ for the benchmark structure

The reaction force exchanged between the two interpolated structural members initially separated by a gap of 0.15 mm being brought into contact is shown Fig. 4.

A modified interpolation rule is required for the material fracture toughness, $G_c$. As presented in Sect. 2.1, when contact occurs, the strain energy of the void material increases to values larger than those experienced in the solid material. The energy balance setup in Eq. (16) could then result in unphysical damage occurring in the void domain. To circumvent this effect a modified rule is utilised to represent $G_c$ given by

18 $$\begin{aligned} G_c(\rho ) = \left( \frac{a}{1+\exp {(b \cdot \rho )}}+\rho \right) \ G_c \end{aligned}$$

**Fig. 5:**
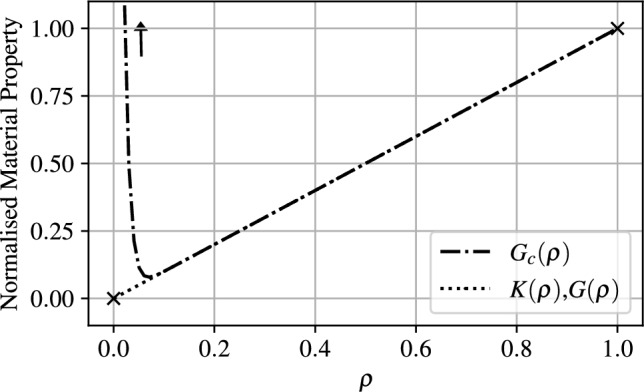
Interpolation rules as a function of pseudo-density $\rho$

With this addition, $G_c \rightarrow a/2$ as $\rho \rightarrow 0$, and *b* controls the slope of the curve. Similar to $\gamma _0$, *a* and *b* must be chosen to ensure stable numerical solutions while avoiding damage from occurring in void material. A comparison of the interpolation used for *K* and *G*, and the augmented interpolation shown in Eq. (18) used to define $G_c$ are depicted in Fig. 5 for $\gamma _0 = 1 \times 10^{-5}$, $a=10$ and $b=100$.

For discrete structures only the limit values ($\rho = 0$ and $\rho = 1$) of these functions are considered in the solution. Although not strictly required for the current application, these interpolation rules could be beneficial for applications where intermediate densities are encountered. This extends the solution validity for graded material and allows for the design of problem-specific interpolation function, provided that the function limits are tuned correctly.

## Numerical implementation

**Fig. 6:**
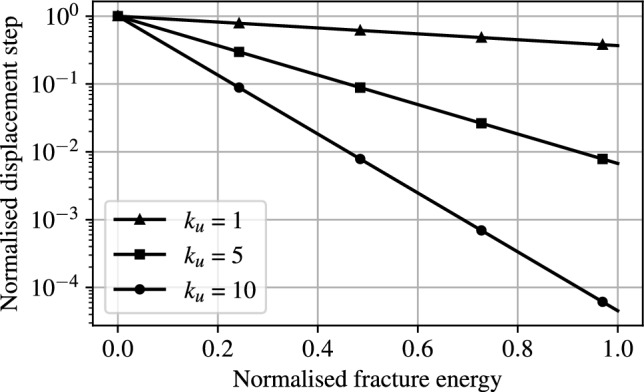
Influence of the parameter $k_u$ on the predicted displacement steps

The open-source finite element (FE) library Firedrake [50] is used to solve the mixed, nonlinear problem described in Eq. (16). To solve the material fracture-contact problem, we adopt an augmented Newton solver. At each imposed displacement the solution of Eq. (16) is searched by setting $\mathcal {H}_n = \mathcal {H}_{(n-1)}$. Provided the displacement increments are sufficiently small, this approach offers a close approximation to a fully coupled solution and can be interpreted as a single-pass staggered solution strategy. This approach is found to improve stability and computational efficiency. Conventionally, the solution procedure to fracture problems involves reducing displacement increments arbitrarily to very small values when fractures propagate. However, this method requires prior knowledge of when fractures occur. As the goal is to utilise this framework in applications where structural boundaries and crack paths are not known a priori, we do not utilise this approach. While it is possible to define uniformly small displacement steps instead, this would lead to a significant and unnecessary increase in computational expense. To overcome these issues, a custom, adaptive time stepping scheme is proposed. To balance computational efficiency, stability and accuracy during crack propagation, this scheme adaptively adjusts displacement increments in response to evolving fracture behaviour. It aims to reduce displacement step sizes when fractures are imminent or developing, and to allow larger steps when the system evolves smoothly. A regularized fracture energy indicator, $W_f(\alpha )$ is introduced to track the fracture state of the system defined as

19 $$\begin{aligned} W_f(\alpha ) = \int _\Omega G_c \left( \frac{1}{l_0}\alpha ^2 + l_0 |\nabla \alpha |^2 \right) d\Omega \end{aligned}$$

where $W_f$ quantifies the fracture energy present in the domain at each load step and it is used to inform the size of displacement increments. The density field defining the structural boundaries is interpolated at the onset of the analysis and subsequently held constant throughout the process. The simulation is initialised with a null displacement followed by an initial maximum imposed increment $\Delta u_{max}$. After each successful solution step, the next displacement increment is scaled by a negative exponential function based on the change in fracture energy

20 $$\begin{aligned} u^{i+1} = u^{i} + \Delta u_{max}\exp {\left( -k_u \left( W_f^{i}-W_f^{i-1}\right) \right) } \end{aligned}$$

in which $k_u$ is a tunable scaling factor that governs the sensitivity of the displacement update to changes in fracture energy. A larger $k_u$ results in more aggressive reduction of step sizes during rapid fracture growth, as shown in Fig. 6. The exponential scaling efficiently captures abrupt increases in fracture energy, thereby preventing solution instability and enabling finer resolution during crack initiation and propagation. Conversely, during steady propagation, larger steps are permitted to enhance computational efficiency.

Lastly, to further aid solver convergence, if the solver diverges after a maximum number of nonlinear Newton iterations, the displacement step is halved and Eq. (16) is solved. This behaviour is iterated up to a minimum step size set to a reasonable small value. Following $N_s$ successful load steps the displacement increments are doubled, up to the maximum scaled step size.

## Numerical results

The unified material failure and contact model is validated using two classical results found in fracture mechanics literature, with the added consideration of internal self-contact. The purpose of this study is to assess the integration and mutual consistency of the two models, rather than to improve or refine either model individually.

### Three point bending

**Fig. 7:**
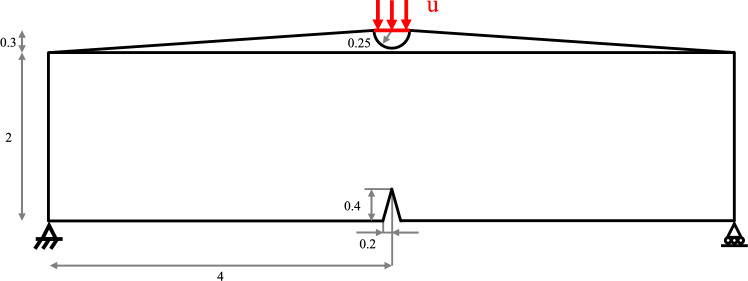
Geometry and boundary conditions for the three point bending validation case (units in mm)

The first example considered is a notched three point bending test. A solid semicircular indenter is placed above the notched specimen with a gap of $l_h = 0.05$ mm as shown in Fig. 7.

**Fig. 8:**
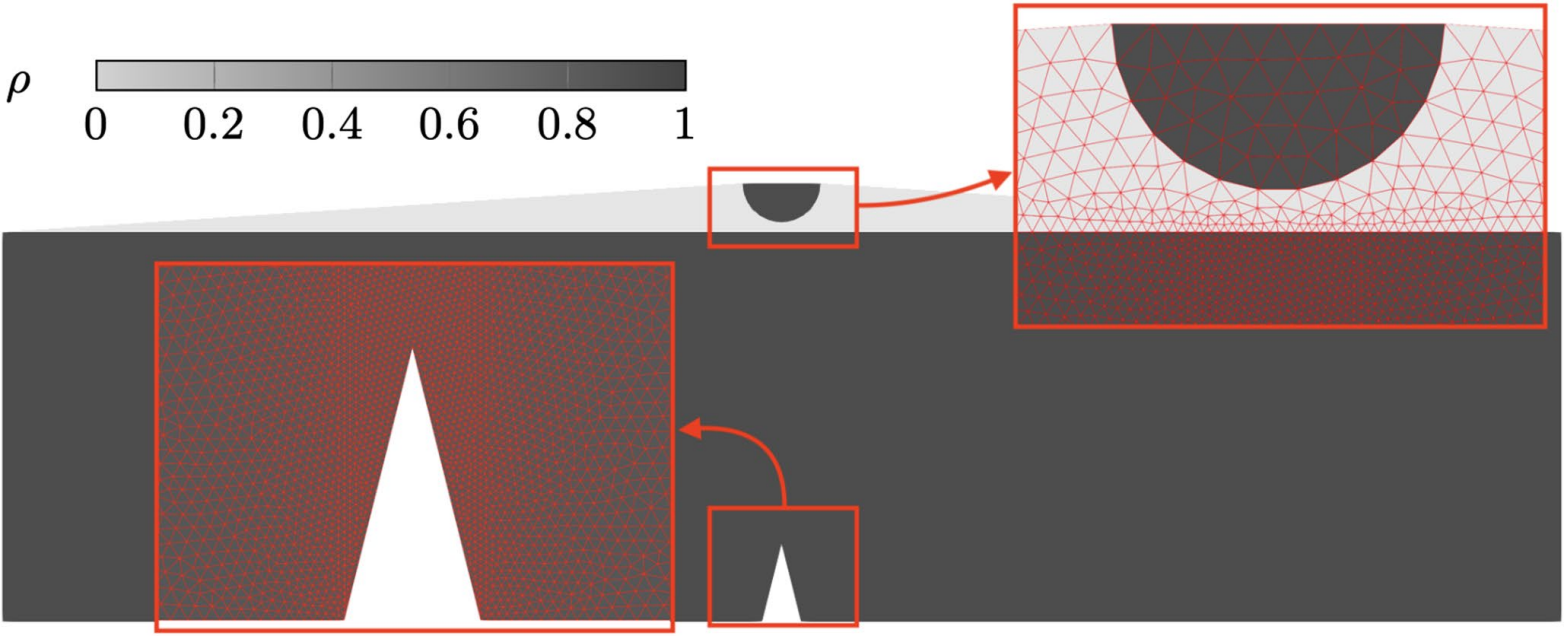
Interpolated density field and adaptive mesh for the three point bending validation case

An adaptively refined mesh is generated, with refinement along the expected crack propagation path and a density field representing the structural boundaries has been interpolated on the domain, as shown in Fig. 8.

The geometry, loading, boundary conditions and material properties are the same as those presented in [8] to allow for comparison and reported in Table 1.

**Table 1:** Material properties for the three point bending test

$E_0$ [MPa]	$\nu$ [-]	$G_c$ [kN/mm]	$l_0$ [mm]	$\gamma _0\,[-]$
$20.8\times 10^3$	0.3	$5.4\times 10^{-4}$	0.03	$5\times 10^{-6}$

**Fig. 9:**
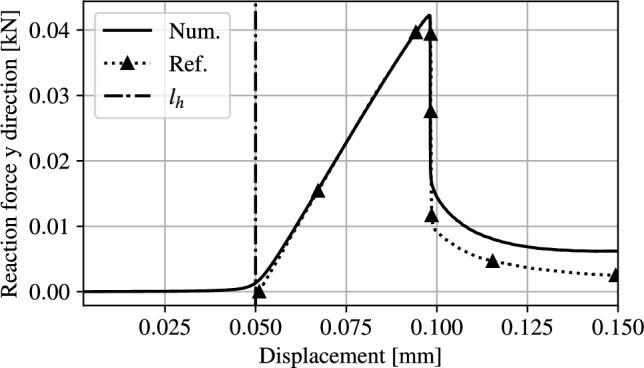
Three point bending test force-displacement comparison with the results in [8]

From the Young’s modulus $E_0$ and the Poisson’s ratio $\nu$, $K_0 = \frac{E_0}{3(1-2\nu )}$ and $G_0 = \frac{E_0}{2(1+\nu )}$ can be computed. Since the relative strains in this example are sufficiently small the results of the TMC hyperelastic model are comparable with those assuming linear elasticity considered as a reference. The minimum refined mesh size $h_{min}$ has been estimated to be $l_0 \ge 3 \ h_{min} \simeq 0.0075$. A void stiffness $\gamma _0=5 \times 10^{-6}$ is found to provide a good balance between solver stability and simulation accuracy. Instead of imposing a displacement on a portion of the top edge of the beam, in this example the displacement is applied to the flat side of the semicircle, as shown in Fig. 7, leading to contact with the notched specimen. A maximum displacement increment $\Delta u_{max} = 0.5 \times 10^{-3}$ mm is applied, up to a total final displacement of 0.15 mm. Finally, the vertical component of the reaction force is evaluated along the top edge of the semicircle (highlighted in red in Fig. 7). The resulting force-displacement curve ("Num."), is compared with the reference results ("Ref.") presented in [8], as shown in Fig. 9.

Since the reference data starts from zero displacement, to ease the comparison, the reference curve has been shifted by $l_h$. As expected, the reaction force remains nearly zero until contact occurs at an applied displacement of $\ l_h$, where a smooth increase in reaction force occurs. The unified contact and failure model is found to closely replicate slope, peak force and displacement of the reference model as well as the maximum force and displacement before failure. Fracture occurs at $u = 0.096$ mm and propagates as expected as shown in Fig. 9. A discrepancy in residual reaction force is observed between the two models. This discrepancy can be attributed to an altered stress state in the indentor. Moreover the residual stiffness evaluated in this example includes both the indentor domain and the void domain in which high energy levels are stored.

**Fig. 10:**
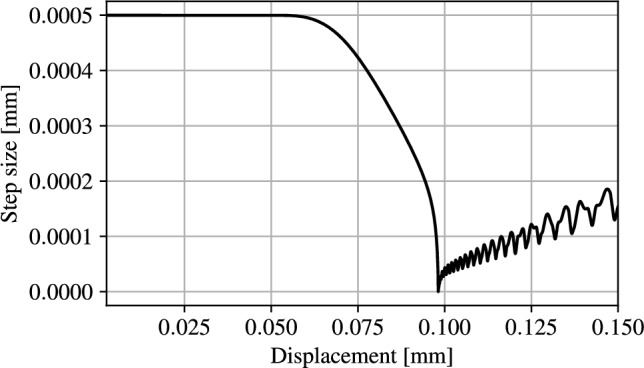
Displacement increment modulation in function of the applied displacement

**Fig. 11:**
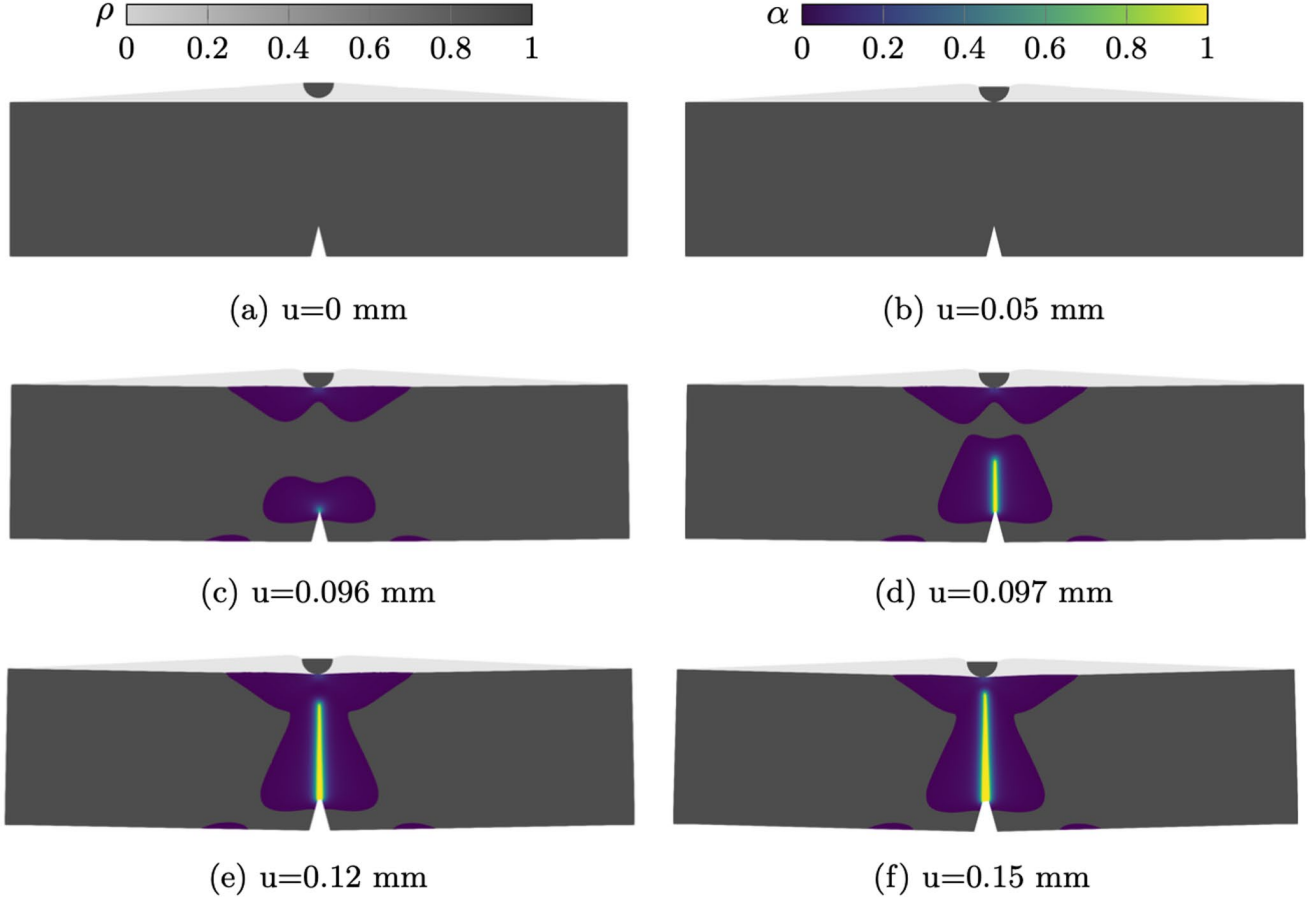
Deformed structure and overlayed phase field at applied displacements

The displacement steps resulting from the adaptive updates from Eq. (20) are shown in Fig. 10, utilising 1000 non-uniform iterations, with a minimum step size of $$1\times 10^{-8}$$  mm with $$k_u = 1000 $$. The displacement increments decrease as soon as contact occurs. This is a direct consequence of the AT-2 fracture model, in which material failure occurs as soon as a load is applied on the structure. In this regime the variation in $$\alpha $$ is small enough to not influence the structural behaviour but large enough to predict the start of the cracking process, impacting the step size. As evidenced in Fig. 11b,where the minimum value of α has been restricted to 0.001 for visualisation purposes, α_max <0.001 when u= 0.05 mm. As the displacement is increased beyond l_h, the displacement step sizes decrease rapidly. Once crack nucleation occurs, the step sizes starts to increase with a final value of 0.000153 mm.

Figure 11 shows the crack initiation and progression overlaid on the structural deformation at discrete applied displacement steps. We note that no damage is experienced in the void material phase due to the critical energy release rate interpolation defined in Eq. (18). The coupled unified model demonstrates strong agreement with the reference numerical results, offering an automated, adaptive displacement control strategy while simultaneously considering both fracture and contact phenomena.

### L-shaped panel irreversibility

**Fig. 12:**
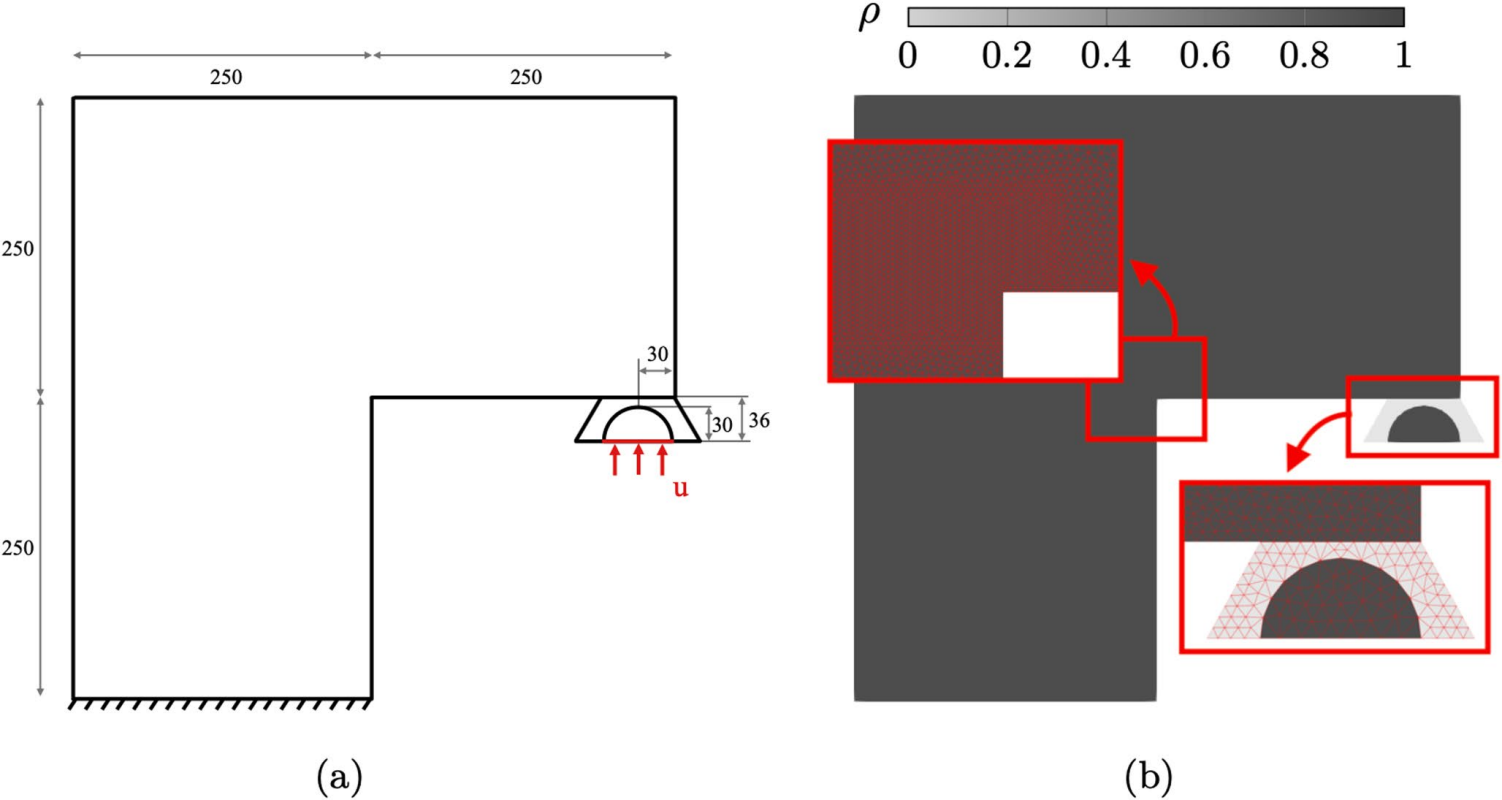
Geometry and boundary conditions (**a**) (units in mm), interpolated density field and adaptive mesh (**b**) on an L-shaped panel

The second example considered is the fracture of a L-shaped beam resulting from contact with an indenter. This example illustrates how the irreversibility constraint enforces the one-way nature of the cracking process. As in the previous example, the domain includes a solid semicircle (indenter) embedded within a void region, as shown in Fig. 12a. To ensure accurate fracture propagation, the mesh has been refined with a minimum cell size of 0.5 mm in regions where cracking is expected, as shown in Fig. 12b.

We replicate the material properties, geometry and simulation conditions adopted in [51] and [8]. The material properties are reported in Table 2.

**Table 2:** Material properties for the L-shaped panel

$E_0$ [MPa]	$\nu$ [-]	$G_c$ [kN/mm]	$l_0$ [mm]	$\gamma _0$ [-]
$2.58\times 10^4$	0.18	0.089	3	$1\times 10^{-6}$

**Fig. 13:**
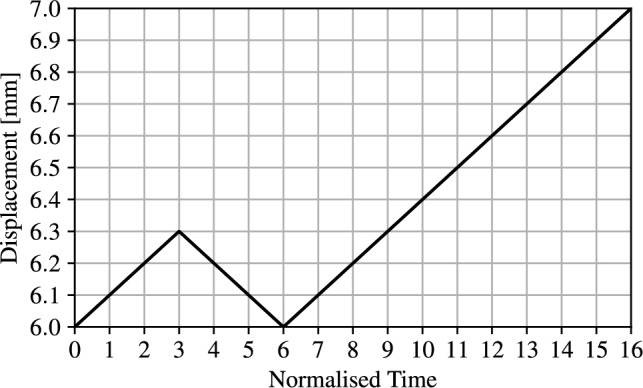
Imposed displacement applied over time to L-shaped panel

We restrict fracture in the indenter and near the fixed edge. Triangular Lagrange elements are adopted to solve Equation (16). No regularisation energy is introduced in the void domain since the void domain is primarily under compression with minimal sliding. As a result, the solver is able to converge without any additional regularisation. Following the setup presented in [51], the displacement is applied in a load-unload-load pattern. Displacement is increased up to $l_h = 6$ mm where contact occurs. The displacement is then increased up to 6.3 mm in the initial loading phase. The displacement is then reduced to $l_h$ (no load), and finally, the displacement is increased in the secondary loading phase up to a final displacement of 7 mm. A graphical representation of the imposed displacement pattern is shown in Fig. 13.

**Fig. 14:**
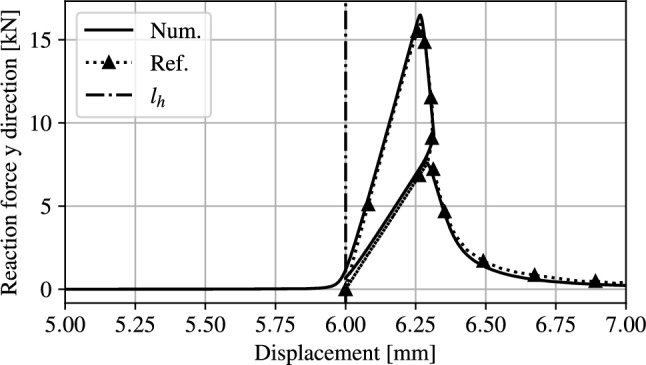
L-shaped panel force-displacement comparison with the results in [8]

**Fig. 15:**
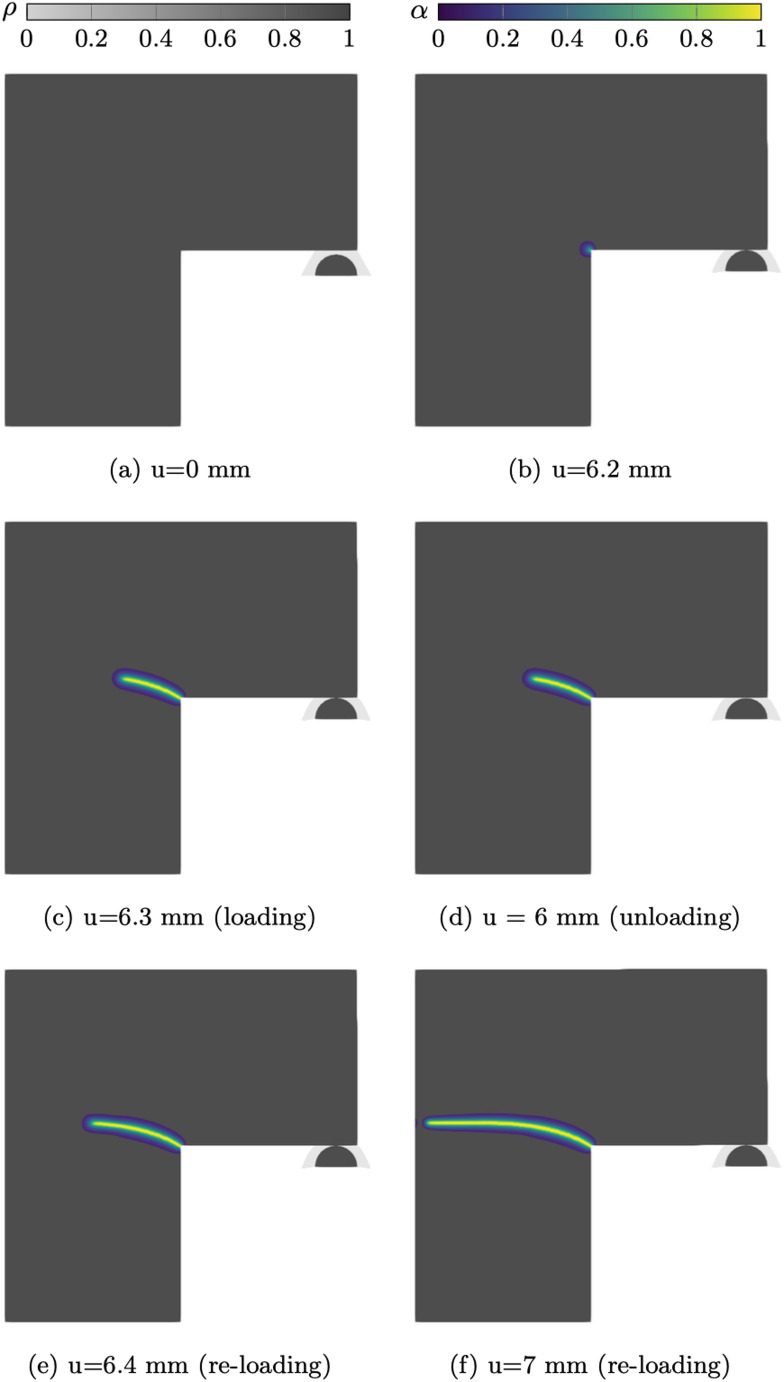
Load history, deformed structure and overlayed phase field for the L-shaped panel

Figure 14 compares the force-displacement curve from the proposed unified model with reference numerical results from [8]. For easier comparison, the reference curve has been shifted to start at $$l_h$$. As expected, the reaction force is nearly null until contact occurs at $$u =l_h$$. Once contact occurs the reaction force increases in accordance with the reference result with a good agreement both in term of peak load and displacement. Since the nature of crack propagation in this case is not as discontinuous as the previous example, the effect of the adaptive time stepping solver is not as fundamental and a $$k_u = 50 $$ is used, but a stable solver is necessary for handling the contact phenomenon. The structural depiction at discrete intervals is shown in Fig. 15, where the minimum value of $$\alpha $$ has been restricted to 0.01 for visualisation purposes and overlaid with $$\rho $$.

As expected, during the unloading phase, no further crack propagation occurs as shown in Figs. 15c and 15d. At the final phase of unloading, at $u=l_h$ the reaction force returns to the original value experienced during the initial loading phase, as shown in Fig. 14.

Finally, during the re-loading phase, the reaction force increases, this time with a smaller slope due to the reduced stiffness, up to a lower peak force of 7.6 kN, in agreement with the reference model, with good agreement in reaction force and maximum displacements. This result demonstrates that the proposed framework is able to accurately capture structural contact without altering the behaviour of the material failure, with excellent agreement to results found in literature.

## Conclusions

The proposed computational framework unifies phase field fracture modelling and third medium contact, enabling variational and differentiable simulation of crack propagation and structural self-contact. A tailored material interpolation model is incorporated to prevent unphysical fractures in void regions, ensuring physically meaningful results. An adaptive displacement control scheme, based on increments in fracture energy, is introduced to improve solver efficiency. This approach accelerates convergence by dynamically reducing displacement step sizes only when necessary, specifically during crack propagation, without requiring any prior knowledge of when or where contact and fractures will occur. Nevertheless, the computational cost remains relatively high, primarily due to the higher-order elements required for regularisation, which can be demanding in large-scale simulations. Rather than seeking to refine either formulation individually, this work focuses on their consistent integration into a unified framework. Two examples were presented to illustrate the capabilities of the model: (1) a three point bending test with the addition of an indenter and (2) a L-shaped beam demonstrating accurate replication of the post failure behaviour through a loading, unloading and re-loading. Although both validations are presented in two dimensions for clarity, the proposed formulation, including the regularisation components, can be extended to three dimensional problems without requiring any conceptual modifications.

The examples show the framework’s capability for handling both irreversible fracture and contact without predefined crack nucleation sites or contact pairs, a significant and useful feature if compared with the models typically presented in literature. This flexibility is particularly valuable for simulating complex failure mechanisms in evolving geometries. In addition, the proposed framework is fully differentiable providing a solid foundation for introduction within a topology optimisation framework. This paves the way for the automated design of structures that intentionally exploit self-contact and fracture as functional design features. This will be the subject of continuing research.

## Data Availability

No datasets were generated or analysed during the current study.
